# The Use of Telemonitoring in Managing the COVID-19 Pandemic: Pilot Implementation Study

**DOI:** 10.2196/20131

**Published:** 2021-09-27

**Authors:** Brian McKinstry, Helen Alexander, Gabriela Maxwell, Lesley Blaikie, Sameer Patel, Bruce Guthrie, Technology Enabled Care TeleCOVID Group

**Affiliations:** 1 Centre for Informatics Usher Institute The University of Edinburgh Edinburgh United Kingdom; 2 Planning & Development NHS Lanarkshire Bothwell United Kingdom; 3 Primary Care Nursing South Lanarkshire Health and Social Care Partnership NHS Lanarkshire Hamilton United Kingdom; 4 Cystic Fibrosis Service NHS Highland Inverness United Kingdom; 5 NHS Education for Scotland Technology Glasgow United Kingdom; 6 College of Medicine & Veterinary Medicine Usher Institute The University of Edinburgh Edinburgh United Kingdom; 7 See Acknowledgments Edinburgh United Kingdom

**Keywords:** telemonitoring, eHealth, COVID-19, primary care

## Abstract

**Background:**

Most people with COVID-19 self-manage at home. However, the condition can deteriorate quickly, and some people may develop serious hypoxia with relatively few symptoms. Early identification of deterioration allows effective management with oxygen and steroids. Telemonitoring of symptoms and physiological signs may facilitate this.

**Objective:**

The aim of this study was to design, implement, and evaluate a telemonitoring system for people with COVID-19 who are self-managing at home and are considered at significant risk of deterioration.

**Methods:**

A multidisciplinary team developed a telemonitoring protocol using a commercial platform to record symptoms, pulse oximetry, and temperature. If symptoms or physiological measures breached targets, patients were alerted and asked to phone for an ambulance (red alert) or for advice (amber alert). Patients attending COVID-19 assessment centers, who were considered fit for discharge but at risk of deterioration, were shown how to use a pulse oximeter and the monitoring system, which they were to use twice daily for 2 weeks. Patients could interact with the system via app, SMS, or touch-tone phone. Written guidance on alerts was also provided. Following consent, patient data on telemonitoring usage and alerts were linked to data on the use of service resources. Subsequently, patients who had either used or not used the telemonitoring service, including those who had not followed advice to seek help, agreed to brief telephone interviews to explore their views on, and how they had interacted with, the telemonitoring system. Interviews were recorded and analyzed thematically. Professionals involved in the implementation were sent an online questionnaire asking them about their perceptions of the service.

**Results:**

We investigated the first 116 patients who used the service. Of these patients, 71 (61.2%) submitted data and the remainder (n=45, 38.8%) chose to self-monitor without electronic support. Of the 71 patients who submitted data, 35 (49%) received 152 alerts during their 2-week observation. A total of 67 red alerts were for oxygen saturation (SpO_2_) levels of ≤93%, and 15 red alerts were because patients recorded severe breathlessness. Out of 71 patients, 14 (20%) were admitted to hospital for an average stay of 3.6 (SD 4.5) days. Of the 45 who used written guidance alone, 7 (16%) were admitted to hospital for an average stay of 4.0 (SD 4.2) days and 1 (2%) died. Some patients who were advised to seek help did not do so, some because parameters improved on retesting and others because they felt no worse than before. All patients found self-monitoring to be reassuring. Of the 11 professionals who used the system, most found it to be useful and easy to use. Of these 11 professionals, 5 (45%) considered the system “very safe,” 3 (27%) thought it “could be safer,” and 3 (27%) wished to have more experience with it before deciding. In total, 2 (18%) felt that SpO_2_ trigger thresholds were too high.

**Conclusions:**

Supported self-monitoring of patients with COVID-19 at home is reassuring to patients, is acceptable to clinicians, and can detect important signs of deterioration. Worryingly, some patients, because they felt well, occasionally ignored important signs of deterioration. It is important, therefore, to emphasize the importance of the early investigation and treatment of asymptomatic hypoxia at the time when patients are initiated and in the warning messages that are sent to patients.

## Introduction

### Background

It is well recognized that some patients affected with COVID-19 who are initially not seriously unwell will later develop severe disease requiring hospital admission. However, in most countries only the most seriously ill are admitted, as hospitals have quickly become overrun [1-3].

It has become clearer that early treatment of people with deteriorating disease is associated with better outcomes [4]. An analysis of early data from Jiangsu province in China suggested that early intervention reduced death rates (<1%) in comparison with Hubei Province (4.3%) where treatment was started later [5]. Likewise, in South Korea, analysis of data showed that later presentation was associated with poorer outcomes, and countries such as Singapore, which had a policy of early admission to hospital, had a very low fatality rate [6,7]. Additionally, delayed admission and level of presenting oxygen saturation (SpO_2_) in English patients has been shown to predict outcome, with even relatively small reductions of SpO_2_ of 95% and below being associated with increases in mortality [8].

Early treatment is effective. Most of the lung injury in COVID-19 is due to inflammation [9], and in severely ill patients, the use of oxygen, steroids, and novel anti-inflammatories, along with general supportive therapy, has been shown to reduce death rate or shorten admissions [10-13].

The elderly and those with underlying medical conditions are at increased risk of deterioration [14]. Other groups (ie, health care staff, some ethnic minorities, and people with high BMI) are particularly known to delay presentation, which is associated with poorer outcomes [15].

The high death rate in the United Kingdom among those admitted too late for treatment to be effective led to calls for more active monitoring, both to detect early deterioration in these at-risk groups and to encourage them to seek help [16].

### Detecting Early Deterioration

Detecting deterioration can be challenging. Many patients present with pronounced arterial hypoxemia, yet without proportional signs of respiratory distress or sense of breathlessness. Dyspnea was reported by only 18.7% of hospitalized patients in one series [17]. However, in some patients with significant lung disease, normal SpO_2_ can also be initially maintained by hyperventilation. It is important, therefore, to consider symptoms of both breathlessness and SpO_2_ in detecting deterioration in COVID-19 [18]. Additionally, in some people with chronic lung disease, borderline SpO_2_ is relatively frequent and may be less predictive in COVID-19 than in the general population [19].

A recent Delphi exercise based in UK primary care, which involved 72 clinicians, set out to develop an early warning score for deterioration in COVID-19 [20]. The authors suggested that the following factors would be valuable in predicting deterioration: fast pulse rate; shortness of breath or respiratory rate; trajectory of breathlessness; pulse oximeter reading, with brief exercise test if appropriate, or symptoms suggestive of hypoxia; temperature or fever symptoms; duration of symptoms; muscle aches; new confusion; being on the shielded list; and known risk factors for poor outcome. They suggested a scoring system, the sensitivity and specificity of which is yet to be assessed.

Many of the physiological parameters above are easily measured by low-cost devices; however, it is important that these meet a quality standard (eg, the International Organization for Standardization [ISO] standard ISO 80601-2-61:2017 for pulse oximeters). These are accurate within the range required to detect desaturation requiring hospitalization. Many wrist-worn oximeters and smartphone-based oximeters are generally unreliable [21-23]. Raised respiratory rate, a strong predictor of poor outcomes, is more challenging to measure remotely [24,25]; however, recently, pulse oximeters that can estimate respiratory rate using the photoplethysmography waveform and its amplitude variation have become available [26,27].

Some countries have recommended and variably implemented the use of self-monitored pulse oximetry with daily telephone follow-up by nurses in a “virtual ward” arrangement [28,29]. However, at times of high community incidence, when demand on all health care services can rapidly rise, such intensive follow-up may be infeasible given that most patients will remain relatively well.

### Telemonitored, Supported Self-management for COVID-19

An alternative is to support self-management with a telemonitored approach. Patients are requested to regularly record symptoms and physiological parameters and, if these suggest deterioration, automatic alerts to the patient recommend seeking advice or urgent care. The record is available for review by their clinicians. This is expected to facilitate early intervention and, hence, improve the patient’s eventual outcome.

Telemonitoring has been adopted in several locations worldwide. As yet, there are no randomized controlled trials (RCTs) of telemonitoring in COVID-19, although two are underway in the United States and Norway and are scheduled to report results later in 2021 [30,31]. However, several papers describing the early experience with telemonitoring systems in COVID-19 [32,33] and facilitation of early hospital discharge after being hospitalized with COVID-19 have been published [34-37]. All made use of pulse oximetry, and some also measured temperature and recorded a variety of symptoms. The implementations employed a range of trigger alert levels for SpO_2_ (from <90% to <95%). The number of alerts varied across the studies, reflecting the trigger-level settings and different populations being monitored: some were relatively young with few underlying conditions, whereas in one study, some patients were receiving home oxygen [38]. Overall telemonitoring was perceived as being helpful in detecting deterioration.

Ideally, telemonitoring systems should work across a range of mobile phones, tablets, and computers, and they should link to health service systems using open standards so that the service obtains timely robust data, which are critical to managing workload. Telemonitoring systems that require patients to subscribe using their own smartphones or tablet PCs could exclude more vulnerable people, such as older people and those experiencing more poverty, who are less likely to have a smartphone or internet access [39].

There are potential risks to telemonitoring, such as overreliance on physiological parameters by inexperienced clinicians, poor adherence to self-monitoring, failure to respond to alerts, or faulty equipment. Implementations should be within an evaluative framework that examines impact on workload, utility to clinicians, usability, acceptability to patients, and equity of access. In particular, rapid feedback of evaluation findings will be needed to modify and optimize the intervention. Below we describe the design and initial evaluation of a Scottish COVID-19 home monitoring system.

### Scottish COVID-19 Home Monitoring System

In Scotland, health services are provided free at point of care and are paid for from general taxation. Early in the pandemic, a COVID-19 clinical pathway was developed to manage patients according to their level of perceived risk (Textbox 1). Substantial numbers of people, with mild disease at first assessment but potentially at risk of future deterioration, were asked to remain at home and to call back only if symptoms worsened. However, some may have delayed or developed low SpO_2_ with few symptoms and, as a result, may have been admitted to hospital later than was optimal. Recognizing the need for early detection of deterioration in COVID-19 in the late summer of 2020, the Scottish Chief Medical Officer called for systems to detect and manage this.

**Risk stratification of patients suspected of having COVID-19 in the United Kingdom.**

Risk stratification in the United Kingdom involves multiple layers of decision making:

1. People who consider themselves to have an immediately life-threatening illness can phone 999 for emergency ambulance, paramedic assessment, and admission to hospital.
2. People with less severe symptoms are steered to online advice (eg, NHS [National Health Service] 111 Online), where a symptom checker directs people to self-management advice if they have minimal or no symptoms, to call NHS 111 if they have more significant symptoms, or to call an ambulance if they have life-threatening symptoms.
3. Anyone can ring NHS 111 for nonmedical telephone advice and, depending on symptoms and their individual circumstances, a proportion are referred for general practitioner (GP) telephone consultation or emergency assessment (ie, calling an ambulance to attend the emergency room).
4. GP telephone consultation may lead to advice only, face-to-face community assessment, or emergency assessment. Video consultations may also form part of a wider strategy of remote care for COVID-19 [17].
5. Face-to-face assessment may lead to advice to continue self-care at home or to admission to hospital.

### Developing the Monitoring System

An expert group was formed, which was drawn from Scottish Government clinical advisors; primary and secondary care; the Scottish unscheduled care service, NHS (National Health Service) 24; and the Scottish Ambulance Service. A clinical protocol, based on current evidence and early international experience of telemonitoring, was then developed. This protocol was subsequently approved by national professional groups. The system, based on a commercial platform, Inhealthcare, provides twice-daily reminders to record symptoms and collect data on pulse oximetry at rest and postexercise and on temperature over a 14-day period (Textbox 2) [40]. Patients can interact with the system via internet, an app, or SMS, or by responding on their telephone keypad to prerecorded questions.

If responses suggest moderate deterioration, patients receive an automatic message advising them to phone 111, the UK unscheduled care number, and their call is directed to general practitioners for initial telephone assessment. If symptoms or readings suggest severe deterioration requiring possible hospitalization, patients are directed to call 999, the UK emergency number (Multimedia Appendix 1).

**Data collected by the telemonitoring system; data were collected twice daily for 14 days.**

Symptom data:

- Breathlessness—at rest or on minimal activity
- Cough
- Fever
- Severe recent-onset fatigue
- Myalgia (the system is triggered to give advice on self-management only)

Physiological parameters:

- Pulse rate, oxygen saturation (SpO_2_; after 20 minutes seated and, if physically able, after 1 minute walking, or sitting to stand), and temperature

### Setting Triggers for Symptom and Physiological Measurements

Initial alert levels were based on expert clinical judgment and on extrapolation from other respiratory conditions and on national advice [41]. Trigger alerts were set for SpO_2_, pulse, temperature, worsening breathlessness, and severe fatigue of recent onset; see Table 1 for triggers, rationale for these, and advice given to clinicians on how to respond to them. It was expected that linkage of telemonitoring data to outcomes (ie, reassessment, admission to hospital, need for respiratory support or intensive care unit, and death) would inform subsequent adjustment of alert thresholds. Saturation triggers were, in part, relative (eg, a sudden fall from a higher level to 95% or 94% triggered an advice call), but a level of 93% or lower triggered an urgent warning. There was considerable debate about the trigger that occurred as a result of a fall to 95% from a higher level, as there were concerns that this would create unnecessary workload. In the end, concerns, particularly about underdiagnosis of hypoxia in people with pigmented skin, led to the adoption of this trigger. To test postexercise desaturation, patients whose resting saturation was 95% or above were asked to exercise (ie, brisk walk or sit to stand) for 1 minute, or as long as they could, and to remeasure their SpO_2_. If this fell below 94% it triggered an alert. Because of the difficulties interpreting readings from people who had existing significant respiratory conditions and long-term lower oxygen levels, this group was initially excluded.

A symptom report of myalgia or cough resulted in an automatic suggestion to consider using symptom-relieving medicine only and did not trigger an alert.

**Table 1:** Alert triggers set for the Scottish telemonitoring system and suggested responses.

Symptom or physiological reading recorded by patient	Advice to patient	Rationale	Considerations for clinician
Breathlessness or difficulty speaking	You seem very breathless; phone 999^a^	Suggests severe illness, but may be anxiety	Normally managed by the Scottish Ambulance Service
Worsening breathlessness or breathlessness on minimal exertion	You seem to be getting more breathless; please phone 111^b^ for advice	Worsening breathlessness is an early sign of severe COVID-19	Speak with patient to confirm decline: Does patient sound breathless at rest? Are they drinking and eating? If patient has an oximeter and their oxygen saturation is ≥94% after 1 minute of exercise and they are otherwise okay, consider continuing observation with safety-netting; if patient does not have a functioning oximeter, consider seeing them to measure saturation and assess respiratory rate
Severe tiredness or exhaustion in the last 24 hours (the system only triggers a call to 111 if no pulse oximeter was available)	Sudden onset of tiredness can suggest a deterioration in your condition; please phone 111 for advice	Severe tiredness is associated with hypoxia	Speak with patient to confirm decline and review oxygen saturation, if available: Have they become more breathless? Are they drinking and eating? Is there evidence of secondary infection? Consider reviewing to check oxygen saturation if they do not have a functioning oximeter
Oxygen saturation <94%	Your oxygen level is very low; please phone 999	Low oxygen saturation may require oxygen therapy	Normally managed by the Scottish Ambulance Service
Oxygen saturation 94% or 95% at rest (the system only triggers an alert if previously higher than 95%)	Your oxygen level is a little low; please phone 111 for advice	May be important if a falling level, particularly if associated with increased breathlessness	Speak to patient to confirm general status and check for increasing breathlessness. If the level has fallen from a previously high level, particularly in the presence of increased breathlessness, this may suggest worrying deterioration and, therefore, consideration of further assessment
Resting pulse rate >100 beats per minute	Your pulse rate is higher than expected; please repeat after resting and if still over 100, please phone 111 for advice	Resting tachycardia suggestive of serious illness	Speak to patient to confirm general status and increasing breathlessness; compare with previous heart rate measures—if relatively stable and close to 100, consider observing; if rising, consider worsening COVID-19, pulmonary embolus, or arrythmia (atrial fibrillation is a common complication of COVID-19)
Persistent fever of >38 °C for more than 5 days	Your temperature has been high for 5 days or more; please phone 111 for advice	Raises concerns about potential secondary infection; increased risk of serious outcome	Speak to patient to confirm general status, increasing breathlessness, chest pain, colored spit, and symptoms of other infections like urinary tract infection (UTI); consider further examination and investigation
One-off fever of >38.5 °C	Your temperature is higher than expected; please phone 111 for advice	Raises concerns of severe illness	Speak to patient to confirm general status, increasing breathlessness, chest pain, colored spit, and symptoms of other infections like UTI; consider further examination and investigation

^a^The number 999 is the UK emergency ambulance number.

^b^The number 111 is for telephone medical advice and triage.

### Selecting Patients for Monitoring

**Figure 1:**
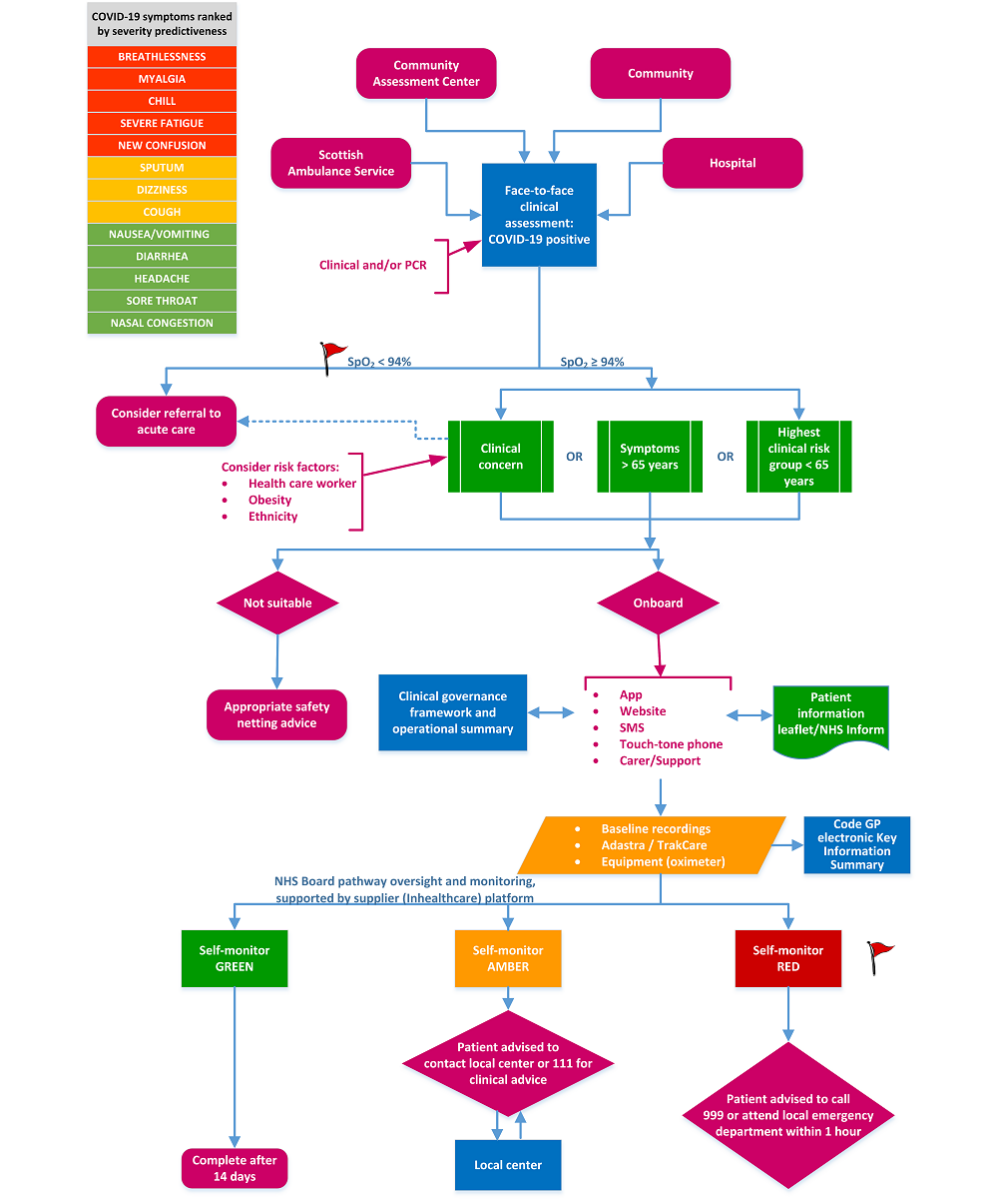
COVID-19 remote monitoring pathway. BAME: Black, Asian, and minority ethnic; GP: general practitioner; HCW: health care worker; NHS: National Health Service; PCR: polymerase chain reaction; SpO_2_: oxygen saturation.

Initially in Scotland, the system was offered to people attending primary care COVID-19 assessment centers in person after a physical examination; it was also offered in the remote and rural setting to patients admitted briefly but considered fit for discharge and self-monitoring. However, it was expected that initiation of monitoring from emergency departments, general practice, or remotely, following video assessment, would also be possible. People considered at higher risk of deterioration, but with symptoms and physiological signs below the threshold for hospital admission, were offered monitoring. Although algorithm-based calculators, such as COVID-AGE [42], were considered, the final decision on whom was considered suitable for monitoring was left to the assessing doctors, and was usually based on age, underlying illness, clinical condition, and capacity to manage the system (Figure 1). Patients were given a pulse oximeter and shown how to use it. They were told not to wait for requests for data if they felt they were deteriorating but to phone immediately for advice. It was made clear to patients that the system was based on self-monitoring, that there was no systematic review of alerts, and that it was their responsibility to seek help if symptoms or physiological measures suggested they should. Patients were also given written guidance on using the device and on what to do should trigger levels be breached; they could also opt to self-monitor without telemonitored support.

A full description of the system, including information for clinicians and patients, governance, and technical information, is available online [43,44].

### Initial Experience With the System

Two Scottish health boards, one rural and one mixed rural and urban, took part in the pilot implementation. Levels of COVID-19 had begun to fall in the rural area; however, the mixed urban and rural area still had high levels of transmission. The experience of the first 116 patients is described in the Results section.

## Methods

Completion of the United Kingdom Research and Innovation, Medical Research Council, NHS Health Research Authority decision tool on April 15, 2021, confirmed that this evaluation “would not be considered research by the NHS” and, therefore, did not require ethical approval. All patients who took up the offer of telemonitoring gave permission for their data to be used to evaluate and improve the service. Data were extracted from the Inhealthcare system and linked to data measuring service resource use by the NHS Board team. The clinical team subsequently obtained verbal consent for a follow-up telephone interview with a sample of patients selected on the basis of age, sex, whether or not they had used the system, whether or not they had received alerts, their response to any alerts, and subsequent resource use.

Interviews were carried out by HA, who was not involved in the design or implementation of the system. Caldicott Guardian approval was granted by NHS Highland on April 15, 2021, for sharing data related to interviewing their patients. This was not required for NHS Lanarkshire, as HA is an employee. All interviews were conducted by telephone (see Multimedia Appendix 2 for interview questions), digitally recorded, and analyzed thematically.

Professionals involved in the implementation were sent a link to an online questionnaire (Multimedia Appendix 2) asking them about their perceptions of the safety and utility of the system, ease of onboarding and explaining the system to patients, the professional user interface, and the appropriateness of the triggers as well as their suggestions for improvement.

## Results

### System and Resource Use Data

Of the first 116 patients who were given oximeters and expressed interest in using the system, 56.0% (n=65) chose to use SMS, 27.6% (n=32) chose to use an app, 6.9% (n=8) chose to use a web portal, and 4.3% (n=5) chose to use automated callback with a touch-tone phone; 5.2% (n=6) of the data were missing. Of the 116 patients who signed up, 71 (61.2%) submitted some data. The remaining 45 (38.8%) patients could choose to self-monitor without telemonitored support. Table 2 shows the demographics of the participants.

**Table 2:** Demographics of the participating patients; 111 patients were from Lanarkshire and 5 were from Highland (N=116).

Characteristic			Patients who submitted readings (n=71)		Patients who did not submit readings (n=45)
**Sex, n (%)**					
	Women	40 (56)		20 (44)	
	Men	31 (44)		25 (56)	
**Age in years**					
	Mean (SD)	51.3 (15.8 )		54.0 (13.2 )	
	Range	24-94		25-87	

The history of alerts and their subsequent service contacts are summarized in Table 3. Of the 71 patients who sent data, 35 (49%) received alerts at some point, logging 152 alerts. Of these 35 patients, 28 (80%) received red emergency alerts, suggesting they call an ambulance, and 7 (20%) patients received amber advice-only alerts. The same episode could trigger several alerts for different parameters or symptoms. A total of 67 red alerts were triggered by SpO_2_ levels ≤93%, and 15 red alerts were triggered by patients responding that they were “unable to speak in sentences because of breathlessness.” Table 3 shows how these patients subsequently used health services. There was one death; however, this occurred 2 days after assessment in a patient who had not used telemonitored support.

**Figure 2:**
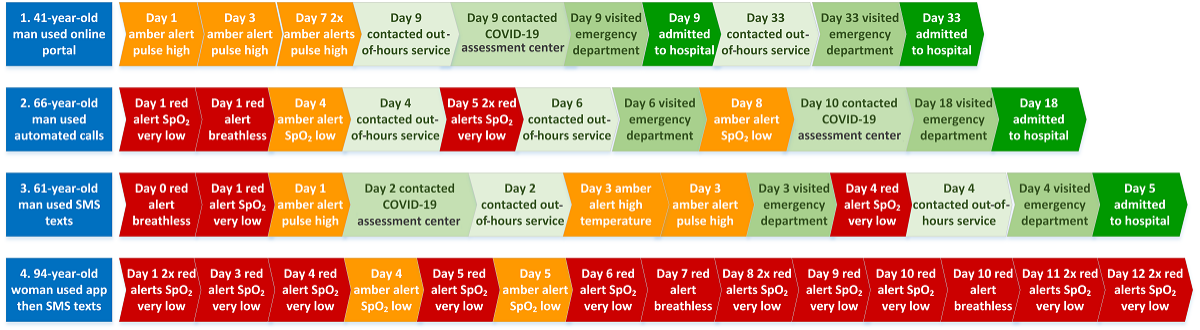
Patient #1 is a 41-year-old man who is asked to call for advice several times because of a high resting pulse rate, but does not do this for 9 days. He eventually contacts the out-of-hours service (phone 111) and is seen at the Acute Respiratory Illness Centre, which handles COVID-19 cases in the community; he is then sent on to the hospital emergency department and admitted to hospital. Patient #2 is a 69-year-old man who, the day after starting monitoring, develops breathlessness and oxygen saturation (SpO_2_) of 93%. He does not call, but his SpO_2_ improves to 94% the next day and he discusses this with the out-of-hours doctor. The following day his saturations fall to 92% and 93%; he is seen in the emergency department but is sent home. One week later he seeks assistance without a trigger warning and is admitted to hospital for one day. Patient #3 is a 61-year-old man who, immediately following assessment, reports shortness of breath and is unable to complete sentences. He ignores this, possibly because he feels no different than when he was examined and thought fit to go home; however, the next day his SpO_2_ drops to 90% and his resting pulse is 103. He delays until the next day before phoning for advice. He is seen and examined and presumably his saturations have returned to normal. He has two other advice warnings because of his pulse rate and temperature, which he ignores; he then develops an SpO_2_ of 90%, which this time triggers an admission to hospital via the Acute Respiratory Illness Centre and emergency department for 17 days. Patient #4 is a 94-year-old woman with dementia whose family were keen to monitor her but wished to avoid admission if possible. They agreed that they would only consider hospital if her oximetry fell to 90% or below. She survived without admission.

There were several instances where patients ignored red alerts to seek advice. Figure 2 shows the case flow of 4 such patients; this is discussed further in the interview analysis in the Patient Interviews section.

Patients had been encouraged not to wait for a request for data if they thought their condition was worsening. A total of 7 patients who sent data, but had not received alerts, had a total of two emergency department attendances, six out-of-hours contacts, three COVID-19 assessment center contacts, and three hospital admissions, with an average length of stay of 2 (SD 2.6) days. Contact rates and hospital admission rates were similar for people who did and did not use the telemonitoring support.

**Table 3:** Alerts issued and subsequent health service use (N=116).

Alerts and health service use^a^	Patients who submitted readings (n=71)	Patients who did not submit readings (n=45)
Total amber alerts, n	70	N/A^b^
Total red alerts, n	82	N/A
Patients who received at least one amber alert but no red alerts, n (%)	7 (10)	N/A
Patients who received at least one red alert, n (%)	28 (39)	N/A
Patients who phoned 111 (out-of-hours primary care), n (%)	18 (25)	11 (24)
Patients who contacted a COVID-19 assessment center, n (%)	8 (11)	4 (9)
Patients who attended the emergency department, n (%)	17 (24)	10 (22)
Patients admitted to hospital, n (%)	14 (20)	7 (16)
Length of hospital stay (days), mean (SD)	3.6 (4.5)	4.0 (4.2)
Deaths, n (%)	0 (0)	1 (2)

^a^A single episode could generate several alerts and several contacts; for example, a patient with breathlessness could also generate alerts for low oxygen saturation, high pulse, and high temperature. The patient could contact NHS (National Health Service) 24, be directed to the COVID-19 assessment center, and then be directed for assessment in the emergency department before admission to hospital. Some patients were admitted directly to hospital via ambulance, while most passed through the emergency department.

^b^N/A: not applicable; patients who did not submit readings did not receive any alerts.

### Patient Interviews

A total of 14 patients agreed to participate in a brief telephone interview to explore their experience of using the system, to determine why some had not followed advice to seek help, and to determine why others had chosen not to send data; see Table 4 for patient characteristics. These were conducted between 5 and 8 weeks after signing up for remote monitoring and lasted an average of 6.5 (2.3) minutes.

All 11 people interviewed who had used the remote monitoring system described it as “easy” or “straightforward.” Interestingly, in 4 cases (29%), the monitoring had been done on behalf of the person with COVID-19, either because the patient was unable to, due to dementia or special needs, or because someone was better able to engage with the technology on their behalf. For this group, being less digitally literate was not necessarily a barrier to remote health monitoring. Although 3 out of 14 (21%) of the interviewees had not uploaded readings, they had used the pulse oximeter and felt it had been “a good idea” or “a comfort” to them. All 14 people interviewed said remote monitoring provided reassurance or “stopped you worrying,” and they endorsed its use by others in the same position. However, not everyone monitored for the full 2 weeks, with one saying they “just got scunnered [fed up] with it.”

Out of 14 interviewees, 4 (29%) had received alerts from the system but elected not to follow the advice received. Out of these 4 interviewees, 2 (50%) explained that instead of calling 111 or 999 immediately, they had waited 10 minutes, taken their readings again, and found they had gone “back to normal.” One added there was “nothing to panic about,” and the other went on to say, “I knew I wasn’t really needing help.” This was also the prime motivation for the third person—a former health care employee—who did not follow the advice received: “I know myself because I felt OK.” Out of the 4 interviewees who did not follow the advice, 2 (50%) felt the health care resources should have been left for “somebody else that does need it.” The decision not to respond to alerts for the fourth patient was made by her niece who was doing the monitoring. She explained that some were triggered by submitting the wrong readings, while others were triggered when her aunt was “really not good.” The niece was clear that, on the night after being assessed, “she wouldn’t have wanted it anyway, so I didn’t bother,” and they had agreed she would wait to get better.

**Table 4:** Characteristics of interviewees and their alert information.

Patient No.	Age in years	Sex	Channel chosen	No. of alerts and type	Responded to alerts	Hospital admission (length of stay)
1	31	Female	SMS text	2 red, 1 amber	No	No
2	49	Female	App	5 red, 1 amber	No	No
3^a^	47	Male	SMS text	2 red	No	No
4^b^	75	Female	SMS text	1 red	No	No
5	66	Male	SMS text	8 red, 4 amber	Yes	Yes (1 day)
6	54	Female	SMS text	1 red, 3 amber	Yes	Yes (<1 day)
7^b^	25	Male	SMS text	1 red	Yes	No
8	47	Male	SMS text	7 amber	Yes	No
9	55	Male	App	1 amber	Yes	Yes (<1 day)
10	36	Female	SMS text	1 amber	Yes	No
11^b^	92	Female	App	0	N/A^c^	No
12	41	Male	SMS text	No readings^d^	N/A	No
13	70	Male	App	No readings^d^	N/A	No
14	50	Male	SMS text	No readings^d^	N/A	No

^a^The patient was interviewed, but their spouse did the monitoring.

^b^The carer or relative who was responsible for remote monitoring was interviewed.

^c^N/A: not applicable because there were no alerts.

^d^The patient self-monitored but did not submit data.

Although it was more difficult to make contact with the 3 patients who had chosen not to submit data, they agreed to an interview. They all valued having the pulse oximeter and reported that they had used it, either twice a day as directed or more often (eg, “every couple of hours”). One was still using it 6 weeks after having received it, and another had found it so useful they had passed it on to other family members who had tested positive for COVID-19.

In terms of the reasons for not uploading monitoring results to the system, one person had clearly misunderstood that they were supposed to do so. They reported that they were “meant to tell the doctor” and had not been asked to submit results via a mobile or landline phone or computer. They demonstrated a facility with taking their readings during the interview. The other two who had not submitted results said they had felt too unwell to engage with it. One valued “having the meter there” because “you knew the safe limits and it was a comfort knowing you were within those safe limits,” and the other referred to the trigger levels in the leaflet and said, “if I got to that level, I’d obviously have to call the emergency services.”

Many interviewees described how much they appreciated having knowledge of what their monitoring levels should be following their COVID-19 diagnosis. One said it was “an eye opener” because “this disease is going after the respiratory system and that’s the one we need to watch.” Another who was “not a medical person” found it interesting “to understand how things change when you walk about and sit down a wee bit out of breath.” A third had been keen to engage after hearing news about pulse oximeters “being able to indicate that people were beginning to become more unwell without feeling it,” and one suffering from fatigue 7 weeks later still checked their levels after being active.

Curiously, one interviewee who had not responded to their alerts suggested others should behave differently, saying “I would like to think they would do what it says and respond.” Another said that the reassurance they got from monitoring meant they “didn’t phone NHS 24 [111, the unscheduled care service] as much as maybe without it [they] might have,” and one felt more generally that it would “save a lot of people from phoning 111 or 999 when really it wasn’t necessary.”

### Professionals’ Views

A total of 14 professionals responded to the online survey: 6 (43%) doctors, 6 (43%) nurses, 1 (7%) administrator, and 1 (7%) respondent who did not give their role. Out of the 14 professionals, 3 (21%) had not used the remote monitoring system, but one of them commented “it’s a great idea” and explained the only reason they had not used it was because they had mainly seen children rather than adults. One of those who had not used the system did not consider the system to be useful or safe.

Of the 11 professionals who had used COVID-19 remote monitoring, 6 (55%) had found it “fairly useful” and 5 (45%) had found it “very useful.” In total, 5 (45%) thought it was “very safe,” 3 (27%) thought that it “could be safer,” 2 (18%) were not sure about its safety, and 1 (9%) felt it was too soon to say. Of the 10 professionals who had initiated patients on the system, 50% (n=5) found it “very easy” and 50% (n=5) found it “fairly easy”; the 3 (27%) who had used the professional user interface thought it was “easy.” It was suggested that the interface could be visually simpler, and that permission to individualize parameters would be an advantage.

Out of 11 professionals, 7 (64%) felt the trigger levels were about right, 2 (18%) were not sure, and 2 (18%) said that alerts were triggered too early. One of these explained that the information around the levels may need to be expanded, and the other felt that the SpO_2_ level at which calling an ambulance was recommended was too high for many people and would result in too many alerts. In the additional comments section, another felt the number of alerts was “slightly annoying,” and one felt the fact that this was self-monitoring should be stressed to patients and relatives.

### National Implementation

Implementing new systems in the midst of a pandemic is very challenging. This solution faced challenges at local levels in terms of information governance and information technology (IT) compatibility issues, which took much longer than expected to resolve. Despite being a relatively small country, Scotland is divided into 14 health boards, all with their own governance and IT teams across Scotland, which were very stretched with many competing priorities. The solution went live as the peak of Scotland’s second wave had passed, so some areas did not feel the same pressure to prioritize this solution. At the time of writing, four health boards have used the system and another four were preparing to set up the infrastructure to be available in the event of a third wave following ending of restrictions or in the event of a new variant emerging. Other boards wanted to see the result of the pilot before committing to it.

## Discussion

### Principal Findings

In periods where there is high community transmission of COVID-19, health services run the risk of being overwhelmed. It is sensible, therefore, that people with milder illness are managed at home. However, given that some in this group will deteriorate, it is important that deterioration is detected early enough to allow effective hospital treatment. Self-monitoring of symptoms and SpO_2_ provides a means of achieving this.

Some patients are more likely to deteriorate than others and, therefore, selection is important, particularly where resources are constrained. Those “higher-risk” patients selected for home monitoring in the Scottish supported home monitoring system had a relatively high hospitalization rate, suggesting that the selection process was relatively effective.

In general, those patients who opted to use it found supported self-monitoring easy to undertake. It was designed to be accessible, offering both digital and nondigital means of communication. It was interesting that most people opted to interact with the system by SMS, possibly reflecting an older age group. However, marketing research shows that people are highly likely to read and respond to SMS messages, more so than other media, and an advantage is that it will work with all kinds of mobile phones [45].

Clinicians also found the system relatively simple to initiate and were largely convinced of its benefits. However, 39% of patients who were offered the system opted to self-monitor without assistance or not to monitor at all. Patients were introduced to the telemonitoring system at a time when they were variably ill—some felt too ill to use it fully—and when clinical staff were under great pressure. However, everyone interviewed endorsed the system, and those interviewed who had not submitted readings had self-monitored with pulse oximetry. Although patients were also given written information, possibly being approached the following day by phone from a dedicated member of a monitoring team would have allowed a better explanation of the system and encouraged uptake.

Although the patients in our case study who opted for telemonitoring were very positive about the feeling of reassurance it gave them, we found that some ignored serious automatic warnings of deterioration even after receiving clear instructions to seek help. When patients were questioned as to why they did not respond to such warnings, some explained that parameters improved on repeating after a few minutes or that they had miskeyed a response. However, worryingly, others stated that as they felt fine, they did not feel the need to call, clearly not realizing that asymptomatic hypoxia was potentially dangerous. Clinicians, therefore, need to strongly emphasize this danger when onboarding patients, and it should be reinforced by written materials and in the warning messages.

Nonetheless, in many cases where deterioration was identified, this appears to have resulted in appropriate assessment either at a local COVID-19 assessment center or emergency department or in a direct hospital admission. Several people contacted support services about alerts that did not result in change of treatment, although this was relatively infrequent. Those who had oximeters but were not transmitting data had had a similar number of contacts. We do not know if this group differed in terms of the severity of their illness at presentation. Interviews suggest that the reassurance provided by monitoring may have prevented some contacts that might otherwise have occurred. In other telemonitored respiratory conditions, patients have said that such reassurance allowed them to self-manage rather than call for advice [46]. COVID-19 remote monitoring was not designed to alter workload, but the results of ongoing RCTs will hopefully inform whether or not it has an impact on both outcomes and workload. The patients interviewed all endorsed its usefulness to them, whether or not they uploaded their monitoring readings, and this early evaluation adds to the emerging evidence base [29].

As a result of this pilot, messaging to patients has changed, thereby emphasizing the need to contact services if saturations are low even if they feel well; likewise, if symptoms raise alerts, patients are encouraged to call even if saturations appear normal.

### Conclusions

Supported self-monitoring of patients with COVID-19 at home is reassuring to patients, is acceptable to clinicians, and can detect important signs of deterioration. Worryingly, some patients, because they felt well, occasionally ignored important signs of deterioration. It is important, therefore, to emphasize the importance of the early investigation and treatment of asymptomatic hypoxia at the time when patients are initiated and in the warning messages that are sent to patients.

## Appendix

Multimedia Appendix 1 Decision tree for the telemonitoring system.

Multimedia Appendix 2 Patient interview questions and staff survey.

## Abbreviations

ISO: International Organization for Standardization
IT: information technology
NHS: National Health Service
RCT: randomized controlled trial
SpO_2_: oxygen saturation
