# Supplementary figures and images for: The Use of Telemonitoring in Managing the COVID-19 Pandemic: Pilot Implementation Study
Source: JMIR Form Res. 2021 Sep 27;5(9):e20131. doi: 10.2196/20131 (PMC8477907; doi:10.2196/20131)

## Slide 1
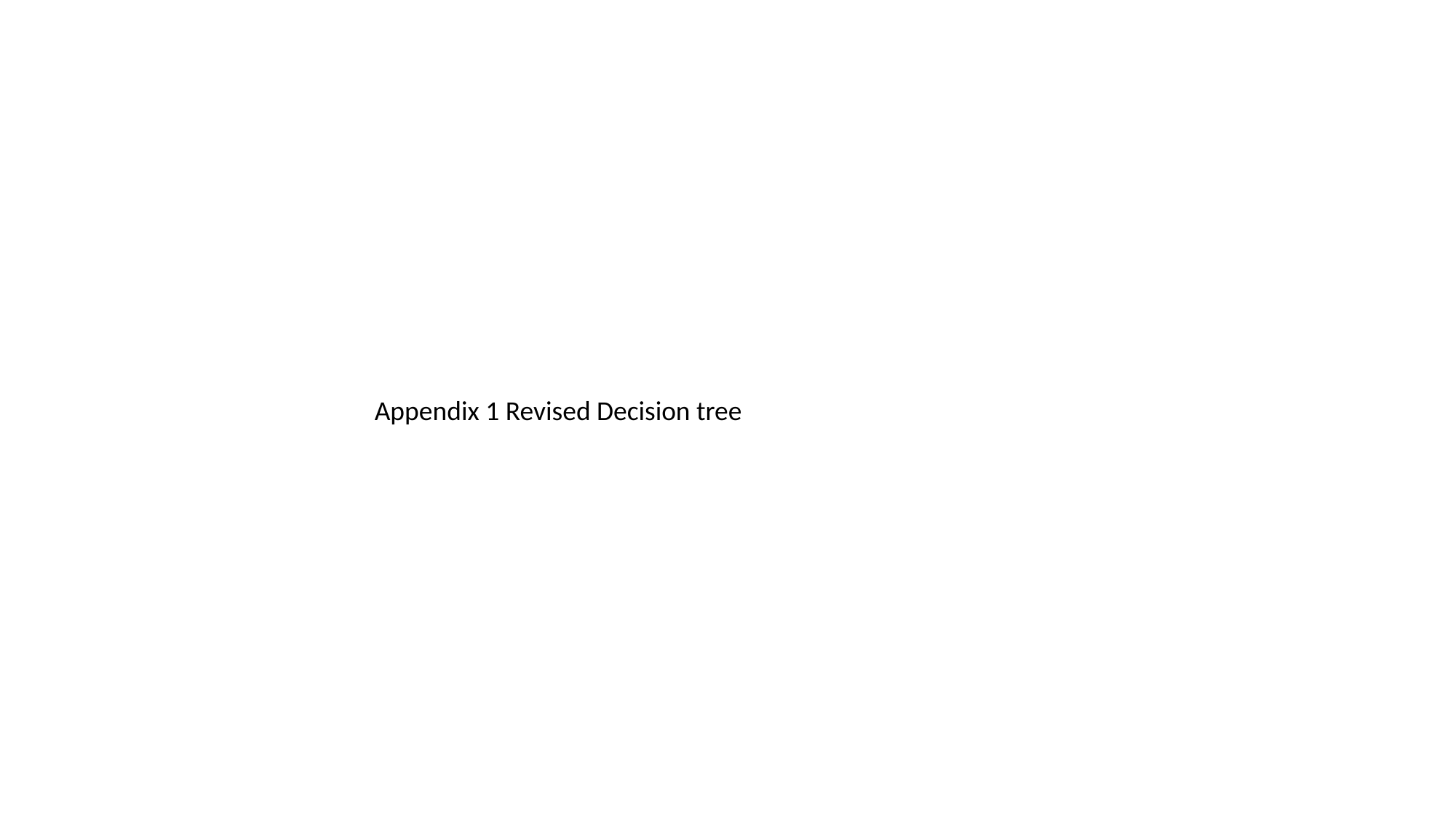

Appendix 1 Revised Decision tree

## Slide 2
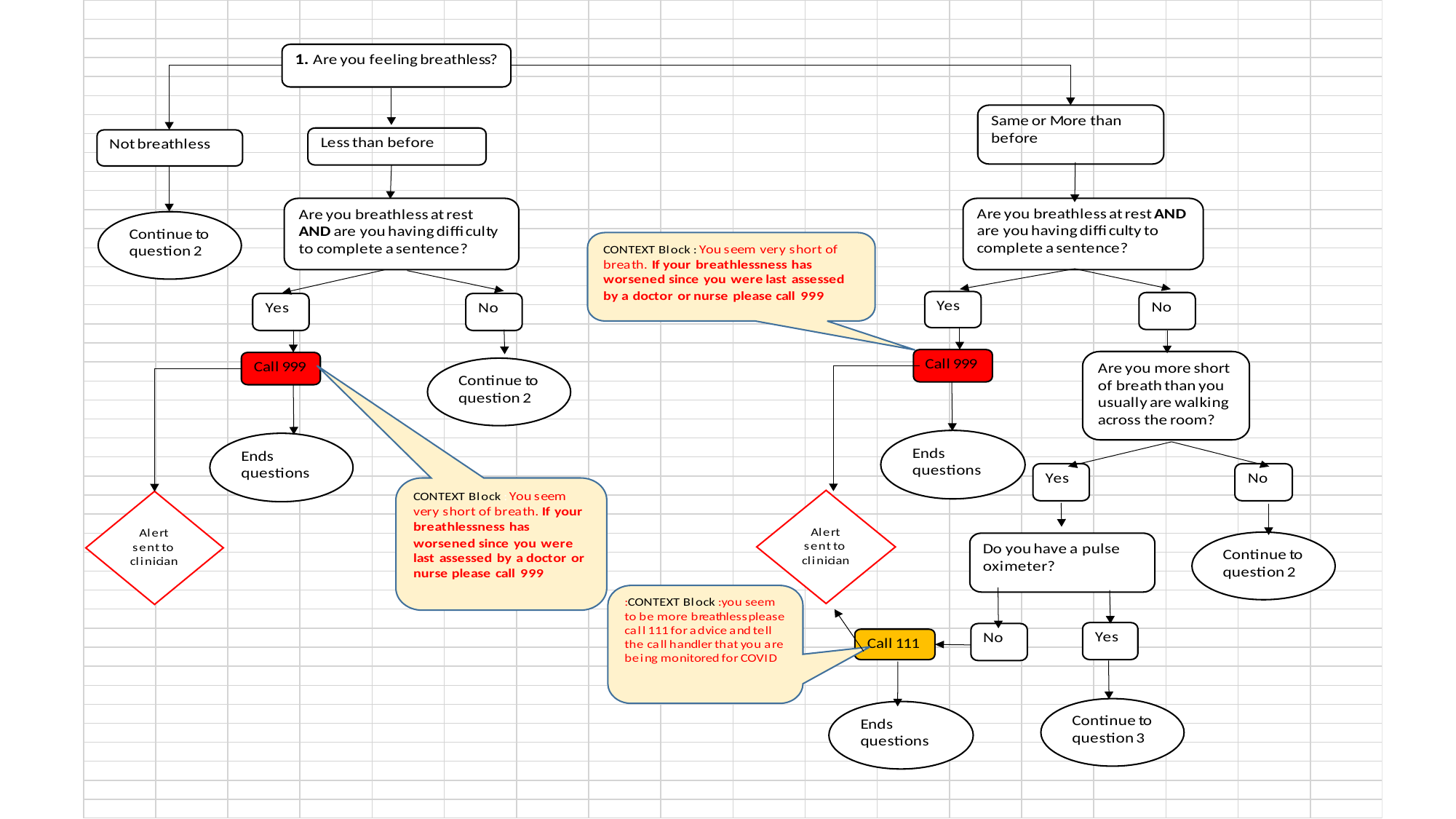

## Slide 3
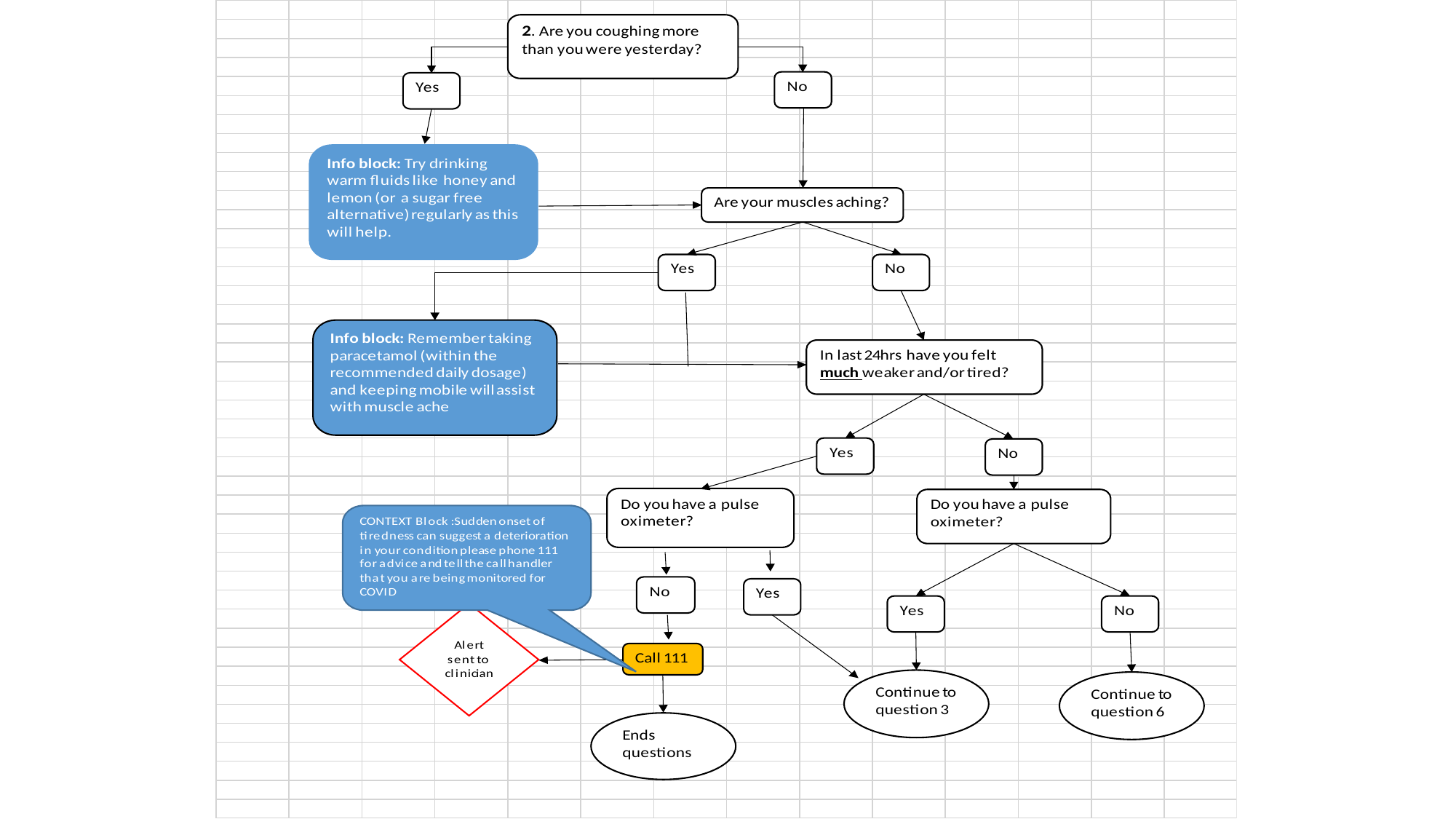

## Slide 4
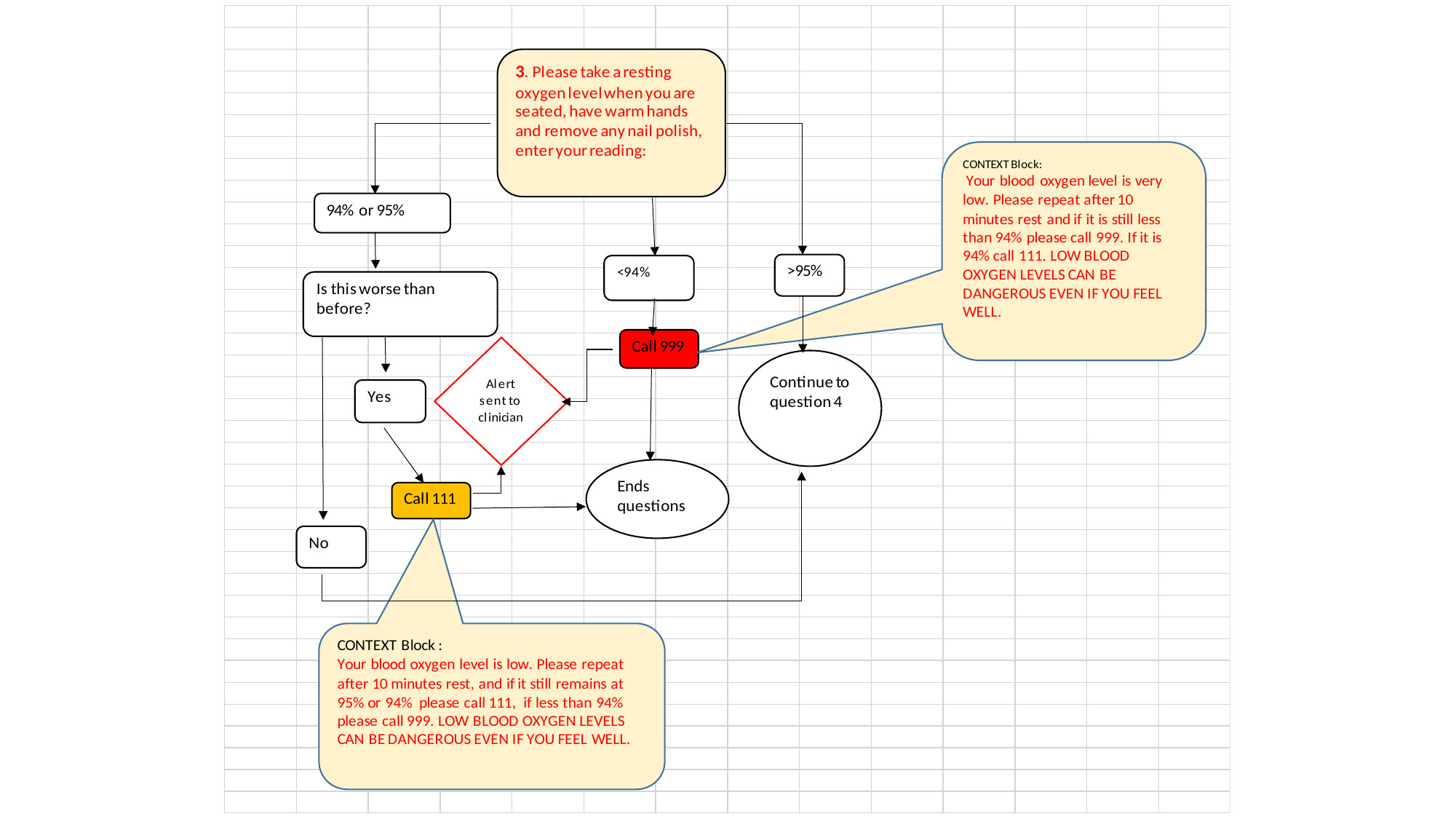

## Slide 5
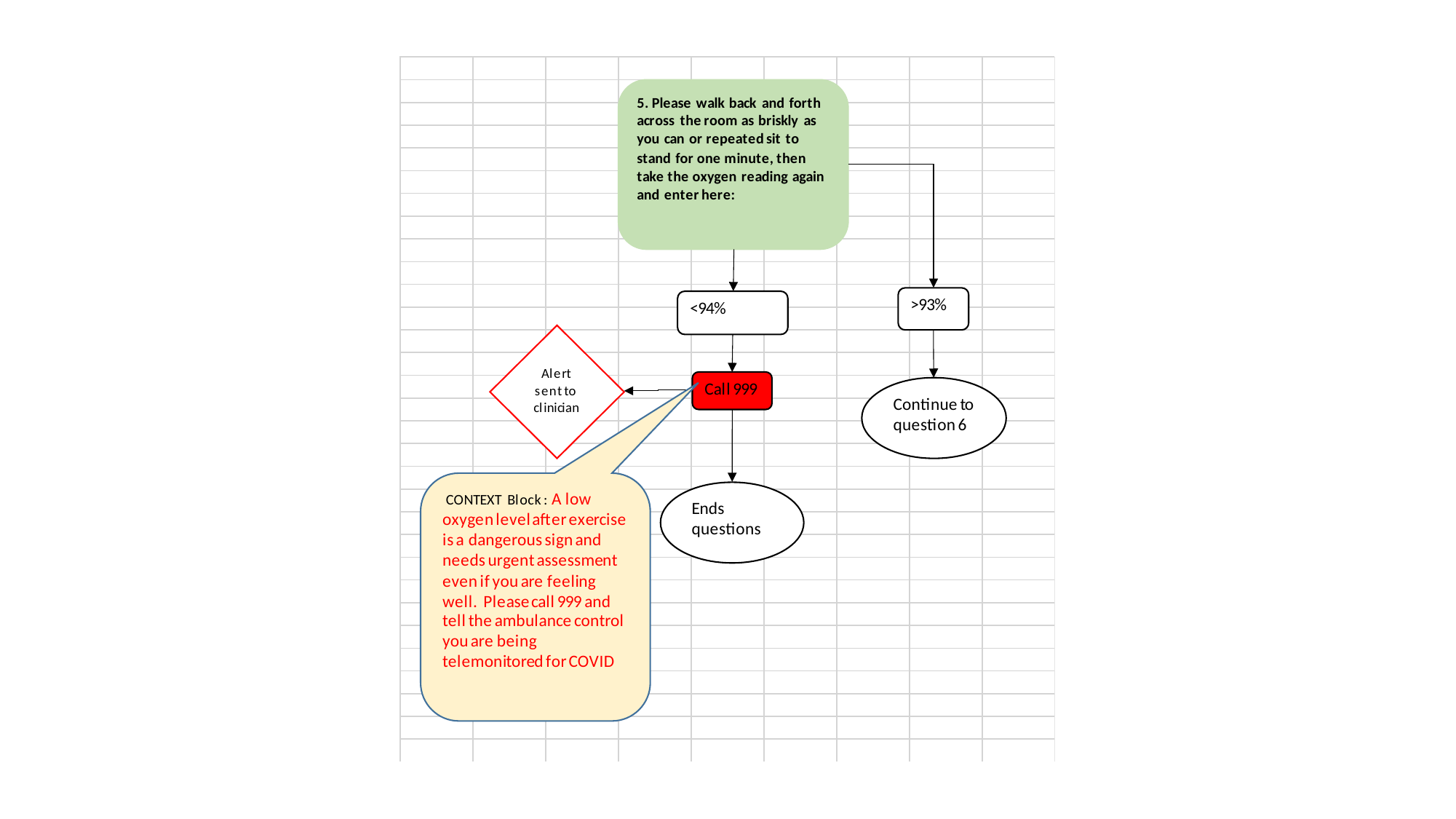

## Slide 6
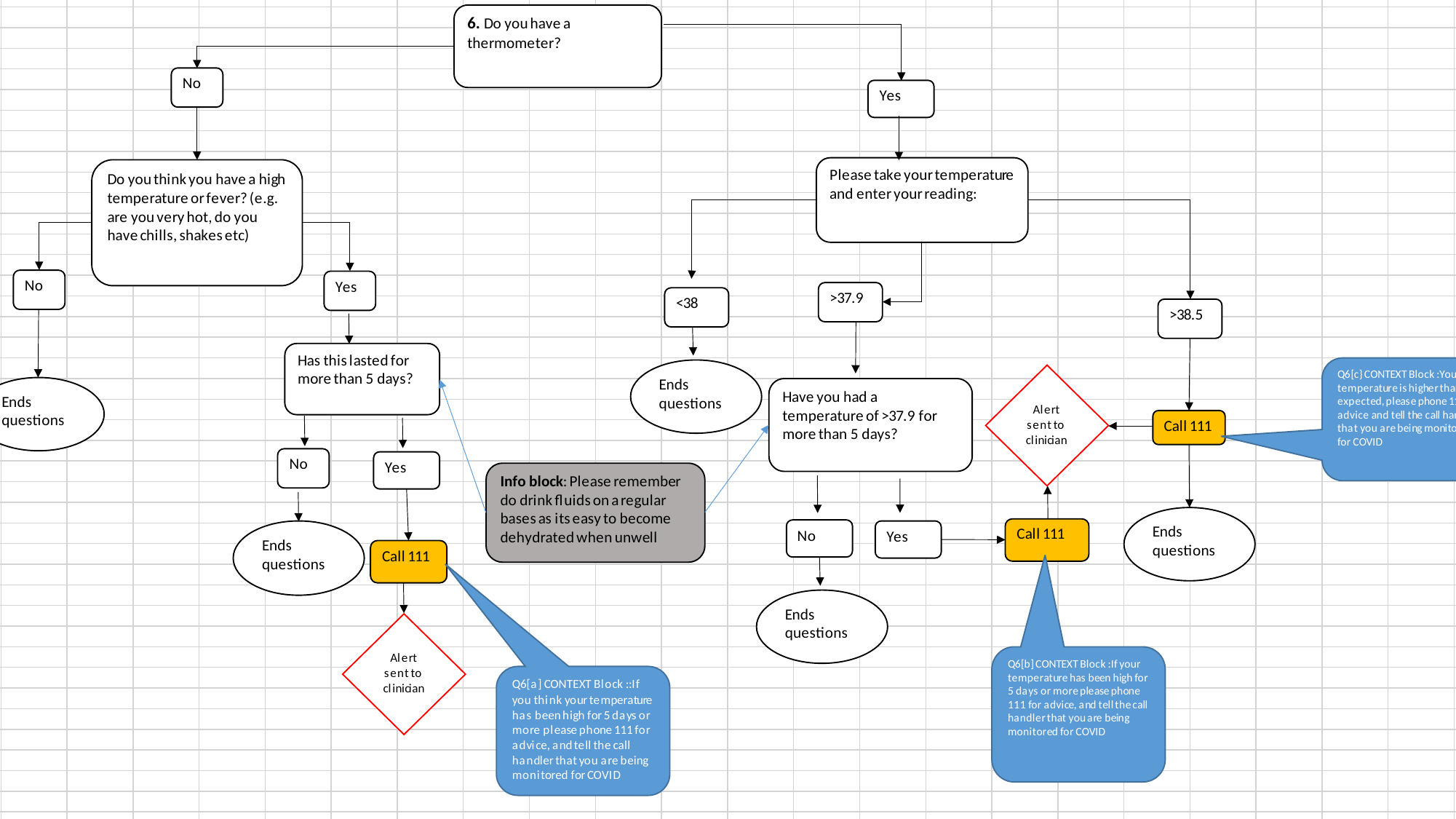

Supplement: Multimedia Appendix 1 [file formative_v5i9e20131_app1.pptx]
