# Supplementary material for: The Use of Telemonitoring in Managing the COVID-19 Pandemic: Pilot Implementation Study
Source: JMIR Form Res. 2021 Sep 27;5(9):e20131. doi: 10.2196/20131 (PMC8477907; doi:10.2196/20131)
Supplement: Multimedia Appendix 2 [file formative_v5i9e20131_app2.docx]

**INTERVIEW QUESTIONS FOR PEOPLE WHO HAVE USED COVID-19 REMOTE MONITORING**

**Q1** I understand you had COVID-19 recently. Can I ask how you are feeling now?

**Q2** Do you remember using the Inhealthcare remote monitoring system to check your symptoms? Which method of monitoring did you choose – text, mobile app, online website or landline telephone? Do you remember why you chose this?

**Q3** Some people say that remote monitoring is reassuring, whilst others find it a bit of a nuisance. How did you feel about remote monitoring when you were first asked to do it?

**Q4** I’m keen to know how you found remote monitoring.

- Did someone show you how to use it at first?

- Was it OK to use or did you have any problem with it?

- Did you get written instructions and, if so, were you able to follow them?

- Were you able to measure your symptoms OK e.g. oxygen, breathlessness?

- How did you find sending in your readings?

- Did you always manage to respond to the questions the system sent you?

**Q5** The remote monitoring system is designed to trigger alerts in response to certain readings. It can send an amber alert suggesting you call 111 for advice or a red alert telling you to call 999. We find some people don’t make these calls.

- While you were monitoring your symptoms did you receive any alerts?

- If you received any alerts, did you usually call 111 or 999?

- If yes, did you find the advice helpful or not? If no, can I ask the reason why you didn’t call?

- Did you seek any medical advice that was not in response to an alert, and why?

**Q6** Based on your experience of remotely monitoring your symptoms, would you recommend it to others who had COVID-19?

**Q7** Is there anything else you want to say about remotely monitoring your COVID-19 symptoms?

**STAFF SURVEY ON THE USE OF COVID-19 REMOTE MONITORING**

**Q1** Roughly how many patients have you used COVID-19 remote monitoring with?

**Q2** What is your role in relation to COVID-19 remote monitoring?

**Q3** In which NHS Board are you using COVID-19 remote monitoring?

**Q4** Have you found COVID-19 remote monitoring useful?

**Q5** Do you feel COVID-19 remote monitoring is safe?

**Q6** COVID-19 remote monitoring is designed to trigger alerts to patients that ask them to call either 111 or 999 depending on the severity of their symptoms. What is your view of the level at which these triggers are currently set? If you think any of the trigger levels are too high or too low, which would you change?

**Q7** How have you found the process of adding patients to the Inhealthcare system for COVID-19 remote monitoring?

**Q8** How do find the process of explaining COVID-19 remote monitoring to patients?

**Q9** How do patients respond to being offered COVID-19 remote monitoring?

**Q10** Have you used the clinician interface for COVID-19 remote monitoring?

**Q11** Would you recommend the COVID-19 remote monitoring system to colleagues?

**Q12** Is there anything you have noticed about COVID-19 remote monitoring that you think could be improved?
